# Structural and biochemical analysis of the metallo‐β‐lactamase L1 from emerging pathogen *Stenotrophomonas maltophilia* revealed the subtle but distinct di‐metal scaffold for catalytic activity

**DOI:** 10.1002/pro.3804

**Published:** 2019-12-24

**Authors:** Youngchang Kim, Natalia Maltseva, Mateusz Wilamowski, Christine Tesar, Michael Endres, Andrzej Joachimiak

**Affiliations:** ^1^ Center for Structural Genomics of Infectious Diseases Consortium for Advanced Science and Engineering, the University of Chicago Chicago Illinois; ^2^ Structural Biology Center X‐ray Science Division, Argonne National Laboratory Argonne Illinois

**Keywords:** antibiotic resistance, antibiotics, di‐metal scaffold, infectious diseases, metallo‐β‐lactamase

## Abstract

Emergence of Enterobacteriaceae harboring metallo‐β‐lactamases (MBL) has raised global threats due to their broad antibiotic resistance profiles and the lack of effective inhibitors against them. We have been studied one of the emerging environmental MBL, the L1 from *Stenotrophomonas maltophilia K279a*. We determined several crystal structures of L1 complexes with three different classes of β‐lactam antibiotics (penicillin G, moxalactam, meropenem, and imipenem), with the inhibitor captopril and different metal ions (Zn^+2^, Cd^+2^, and Cu^+2^). All hydrolyzed antibiotics and the inhibitor were found binding to two Zn^+2^ ions mainly through the opened lactam ring and some hydrophobic interactions with the binding pocket atoms. Without a metal ion, the active site is very similarly maintained as that of the native form with two Zn^+2^ ions, however, the protein does not bind the substrate moxalactam. When two Zn^+2^ ions were replaced with other metal ions, the same di‐metal scaffold was maintained and the added moxalactam was found hydrolyzed in the active site. Differential scanning fluorimetry and isothermal titration calorimetry were used to study thermodynamic properties of L1 MBL compared with New Deli Metallo‐β‐lactamase‐1 (NDM‐1). Both enzymes are significantly stabilized by Zn^+2^ and other divalent metals but showed different dependency. These studies also suggest that moxalactam and its hydrolyzed form may bind and dissociate with different kinetic modes with or without Zn^+2^ for each of L1 and NDM‐1. Our analysis implicates metal ions, in forming a distinct di‐metal scaffold, which is central to the enzyme stability, promiscuous substrate binding and versatile catalytic activity.

**Statement:**

The L1 metallo‐β‐lactamase from an environmental multidrug‐resistant opportunistic pathogen *Stenotrophomonas maltophilia K279a* has been studied by determining 3D structures of L1 enzyme in the complexes with several β‐lactam antibiotics and different divalent metals and characterizing its biochemical and ligand binding properties. We found that the two‐metal center in the active site is critical in the enzymatic process including antibiotics recognition and binding, which explains the enzyme's activity toward diverse antibiotic substrates. This study provides the critical information for understanding the ligand recognition and for advanced drug development.

**Abbreviations**
APS: advanced photon source
CSGID: Center for Structural Genomics of Infectious Diseases
DSF: differential scanning fluorimetry
EDTA: ethylenediaminetetraacetic acid
IPTG: isopropyl‐β‐d‐thiogalactoside
ITC: isothermal titration calorimetry
Km: Michaelis–Menten constant
MBL: metallo‐β‐lactamase
MR: molecular replacement
NDM‐1: New Deli metallo‐β‐lactamase −1
PCR: polymerase chain reaction
RMSD: root mean square deviation
SBC: Structural Biology Center
TEV: tobacco etching virus

## 1 INTRODUCTION

**Figure 1:**
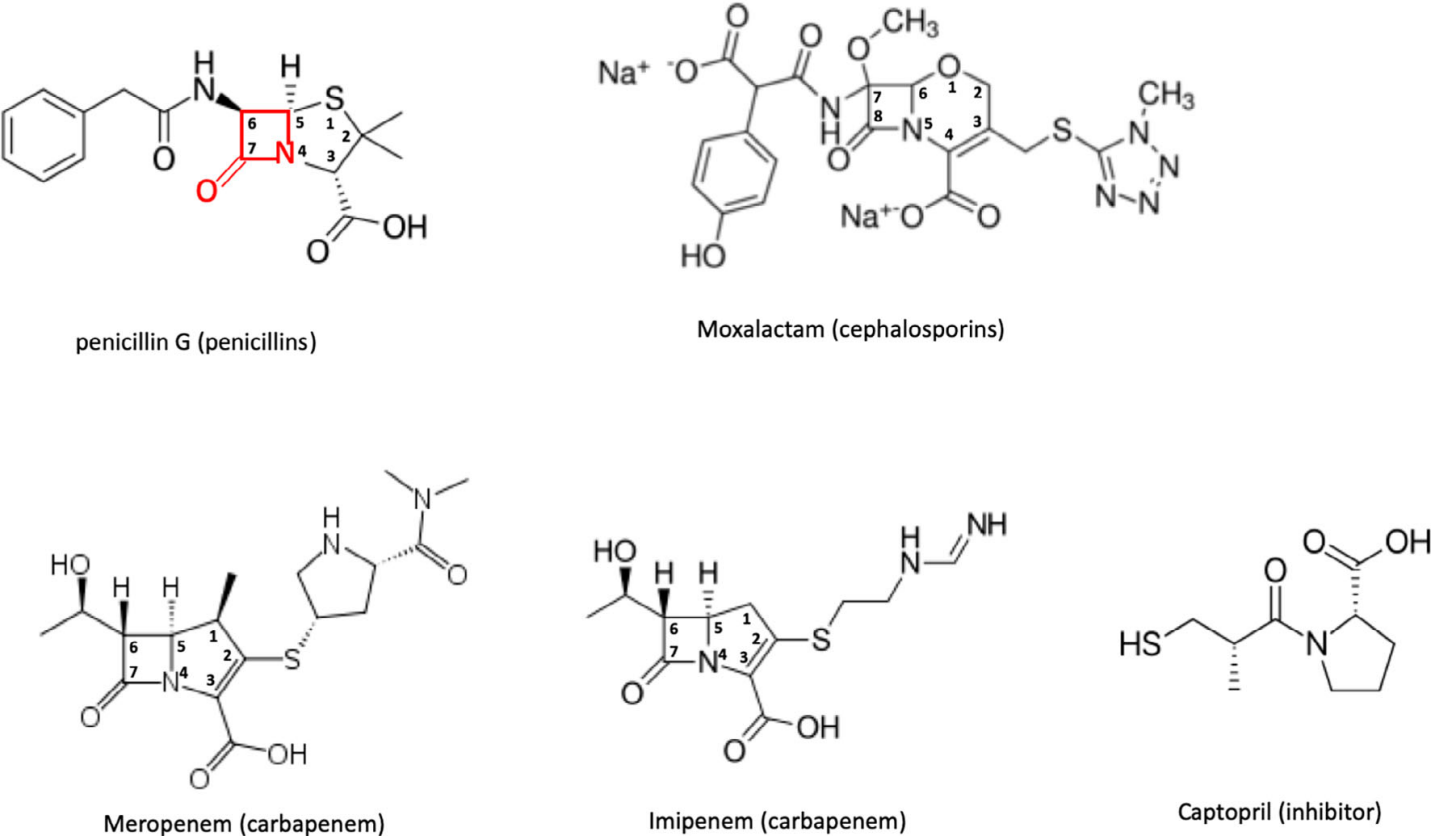
β‐lactam antibiotics and the inhibitor used for the structural study. A β‐lactam is shown in red as a part of penicillin G. The numbering for β‐lactam is also provided for carbapenems. The group names of the antibiotics are shown in parentheses

Since penicillin was first used against bacterial infections, β‐lactam antibiotics have been used to treat a wide range of diseases and further advanced to several classes of drugs. These include compounds with a broad‐spectrum substrate profile such as penicillins, cephalosporins, monobactams, and carbapenems. The β‐lactam containing antibiotics are substrate analogs of bacterial transpeptidases involved in cell‐wall biosynthesis.^1^ They act as inhibitors by blocking the cross‐linking of adjacent peptidoglycan chains and are characterized by a four‐membered heterocyclic β‐lactam ring (see Figure 1). The β‐lactam moiety of the antibiotic binds to the catalytic residues at the active site of penicillin‐binding proteins in the cell membrane of bacteria.^2^

Currently, β‐lactam‐based antibiotics cover more than 65% of all clinically applied antibiotics.^3^ However, unrestrained use of antibiotics, including β‐lactams, resulted in the rise of antibiotic resistance leading to a serious global problem. Mechanisms for resistance include enzymatic modification/deactivation of antibacterial drugs, alteration or modification of bacterial proteins that are targets of antibiotics, and preventing antibiotics from reaching the protein targets by changing membrane permeability to restrict drug entrance into bacterial cells and removing them using efflux systems.^4^ One of the most important mechanism of resistance is enzymatic hydrolysis of antibiotics mediated by the bacterial β‐lactamases. Their genes can be located on mobile plasmids or maintained on the bacterial chromosome and they evolve by mutation in response to the presence of new antibiotics or their derivatives.^5^

β‐lactam antibiotics are produced naturally by many bacteria and are used in interspecies competition against a broad spectrum of Gram‐negative and Gram‐positive bacteria. In response, a number of different β‐lactamase classes (A, B, C, and D) and subclasses evolved and have been deployed by bacteria. Many of these systems have been identified and studied over the years. These functional studies are supported by numerous crystal structures, some determined at atomic resolution. β‐lactamases hydrolyze a four‐atom β‐lactam ring, abolishing its antibacterial properties. Enzymes belonging to classes A, C, and D β‐lactamases are serine‐based hydrolases.^6^ Class B enzymes are different and bind one or two metal ions, typically Zn^+2^, playing a key role in their catalytic activities, as such they are called metallo‐β‐lactamases (MBL). There are three subclasses for MBL proteins, B1, B2, and B3, on the basis of their protein sequence, substrate profiles and especially subtle but significant variations in their active site configurations for two Zn^+2^ ions.^7^ B1 enzymes have two‐metal binding sites consisting of three histidine residues coordinating first metal ion and cysteine, histidine, and aspartic acid binding the second metal ion. There is a bridging water (or hydroxide) molecule binding both metals. In environmental bacteria, MBLs genes are mostly acquired through horizontal gene transfer using plasmids.^5^, ^8^ These bacteria can become antibiotic‐resistant infectious agents and are getting extensive clinical attention.^5^ B2 enzymes are resident enzymes and have a metal site formed by an asparagine or glutamine, and two histidine residues for the first metal site and cysteine, histidine and aspartic acid residues for the second site. However, these enzymes are physiologically mononuclear enzymes as they are active only with single metal present and interestingly the binding of second zinc ion inhibits the enzyme.^9^ For B3, three histidine residues form the first zinc site and two histidine residues and aspartic acid make the second site. Similarly, to class B1, a bridging water/hydroxide molecule is also observed in B3 subclass enzymes. B3 enzymes include both chromosome and plasmid born enzymes. MBLs from B1 and B3 classes are able to hydrolyze nearly every β‐lactam antibiotic, including the most recently developed and serving as last‐resort carbapenems. In contrast, class B2 enzymes have a narrow‐spectrum carbapenem substrate profile, with poor activity toward penicillins and cephalosporins. Importantly, MBLs are not inactivated by mechanism‐based inhibitors like clavulanate or tazobactam that form an irreversible covalently bonded complex with the enzyme.^7^

Substrate promiscuity of MBL stems from the MBL fold having a large, open binding pocket consisting of multiple flexible loops and a di‐nuclear Zn^+2^ scaffold, which anchors β‐lactam moiety of a broad range of substrates with different substituents.

The catalytic mechanism for MBLs has been studied greatly including L1, CcrA, BcII, and NDM‐1.^10^, ^11^, ^12^ However, it is still not fully established, and includes proposed nucleophilic water molecule, either the bridging water molecule or from bulk solvent activated by the bridging water/hydroxide molecule attacking carbonyl of β‐lactam ring. Also involved could be one or more additional water molecules, and one or both Zn^+2^ ions to stabilize atoms in the β‐lactam ring. It is accepted that the Zn^+2^ ions are acting as a Lewis acid by interacting with the β‐lactam carbonyl oxygen and facilitating nucleophilic attack. The positive charge of metal ions offsets the negative charge developed on the oxygen atom in the tetrahedral intermediate anion. The zinc ion also lowers the pKa of the directly coordinated water molecule.^10^

Apparently, two zinc sites are not same: the first site Zn1, or M1 as a general metal site 1, is a tetradentate (three His residues and a water molecule for both B1 and B3 subclasses) in a tetrahedral configuration and the second site Zn2, or M2, is more variable penta‐ or hexa‐dentate (Cys, Asp, His for B1 or NDM‐1 and two His and Asp for B3 and two or three water molecules for both) in an approximately trigonal bipyramid configuration. This subtle geometrical and chemical difference in MBL enzymes^13^ can be reflected as potential functional diversity in substrate binding and catalytic activity. Having certain metals could be crucial for MBLs or other proteins with a metallo‐β‐lactamase fold^14^ to bind particular substrates, for example, β‐lactam antibiotics, for necessary catalytic activities in a given specific environmental condition. Therefore, understanding the interactions between the protein and metal ion is vital to advancements in developing effective drugs against MBLs, a task that is now recognized as critical.

*Stenotrophomonas (Xanthomonas) maltophilia* is an environmental multidrug‐resistant Gram‐negative bacillus found in aqueous habitat that is an emerging opportunistic global pathogen, particularly among hospitalized patients. *S. maltophilia* infections have been associated with high morbidity and mortality in severely immunocompromised and debilitated individuals such as cancer, cystic fibrosis, and transplant patients.^15^

*S. maltophilia* L1 is a subclass B3 MBL, one of two chromosome‐encoded β‐lactamases (L2 belongs to a class A).^16^ Several crystal structures of L1^17^, ^18^, ^19^, ^20^, ^21^ and other B3 MBLs have been reported in the complexes with hydrolyzed antibiotics and inhibitors, which are supported by multiple biochemical analysis.^17^, ^18^, ^19^, ^20^, ^21^, ^22^, ^23^, ^24^, ^25^, ^26^, ^27^, ^28^, ^29^, ^30^ B3 MBLs share a relatively low sequence identity of 23–35%; however, they are structurally well‐conserved particularly their di‐nuclear zinc active sites. Here, we extend the L1 studies and report several high‐resolution structures of L1, the two zinc bound form (native form), the metal‐free form (apo form), the captopril inhibitor bound form, several complexes with hydrolyzed antibiotics (imipenem, moxalactam, meropenem, and penicillin G) and with different metal ions (Zn^+2^, Cd^+2^, and Cu^+2^). We focus here on the structural basis of how the L1 enzyme hydrolyzes different groups of lactam antibiotics and how the metal ions contribute to enzyme's activity. The roles of metals to the enzyme's biochemical function is analyzed and compared with more extensively characterized B1 class enzyme NDM‐1.

## 2 RESULTS

### 2.1 Crystal structures of L1

**Figure 2:**
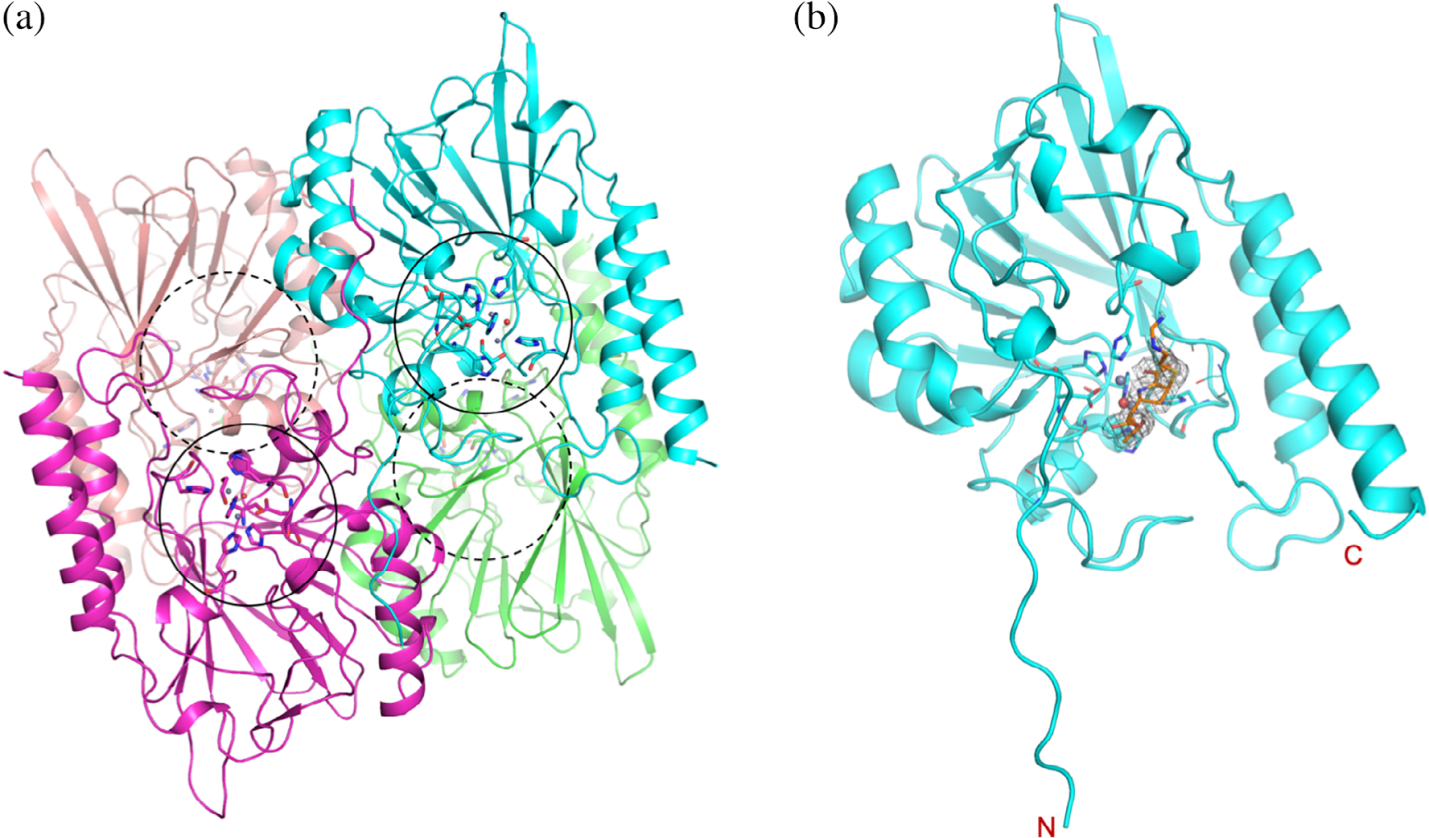
Metallo‐β‐lactamase L1, (a) tetrameric native form and (b) the L1 monomer in complex with hydrolyzed imipenem in approximately the same orientation, the bound ligand is shown in stick drawing caged in the electron density (2fo‐fc map,1 σ), zinc‐binding side chains are also depicted with sticks. The locations of the four active sites in the tetrameric enzyme are indicated by black circles. The dashed circles are for the active sites in the back of the enzymes

The native form structure (L1‐Native) adopts a tetramer of the well‐conserved αβ/βα MBL fold: as previously reported,^5^, ^13^ two layers of a five and a six β‐stranded sheets are sandwiched between four α‐helices, two in each side (Figure 2). The active site/binding pocket is formed between these two β sheets. The protein construct used in the study does not have the first 19 residues of the N‐terminal signal sequence needed for being transported across the cell membrane. The two protein chains exchange the 10‐residue long N‐terminal tails (residues 23–32) to form a dimer with the pseudo twofold rotation symmetry. On the opposite side of the dimer, the other two protein chains form the same dimer rotated 120° along the same twofold rotation symmetry axis and assemble into a pseudo‐symmetric tetramer (or a dimer of dimers) (Figure 2). The tetramer is contained in the asymmetric unit of the P2_1_ space group. Each protein subunit makes extensive contacts with all three other subunits of the tetramer. Interestingly, all other L1 structures described here have only one protein chain in the asymmetric unit in the P6_4_22 space group. The same tetramer is maintained with subunits related by the crystallographic two 2‐fold symmetries that are 120° apart. L1 is the only B class β‐lactamase (BBL) that forms a tetramer and the formation of a tetramer is critical for the catalytic activity and/or substrate profile of L1 as shown by mutational study.^31^ All L1 MBL high‐resolution structures in this study, including the native form (L1‐Native), ligand‐bound forms, and ethylenediaminetetraacetic acid (EDTA)‐treated apo form (L1‐E) are virtually identical to those previously reported L1 structures with or without ligands bound.^17^, ^18^, ^19^, ^20^, ^21^, ^22^, ^23^, ^24^ Root mean square deviations (RMSDs) on 264 or 265 Cα atoms are 0.3–0.4 Å, only a few residues deviate more than 1.0 Å (<2.0 Å) are at the surface of the protein away from the active site. This indicates that the protein is not required to change significantly to bind ligands (hydrolyzed antibiotics, inhibitors, and different metals) in various crystalline conditions. For a note, all the previously reported L1 crystal structures are of L1 from the different strain (type 3) with 88% sequence identity to L1 (*S. maltophilia K279a*) used in this study.

### 2.2 Active site

As shown in the reported structures,^17^, ^18^, ^19^, ^20^, ^21^, ^22^, ^23^, ^24^ the active site is made of two Zn^+2^ (or Cd^+2^ or Cu^+2^) ions and the protein side chains coordinating metal ions at the bottom of the binding pocket. All the following atomic distances are observed in L1‐Native. His116 [standard BBL numbering^32^ is used throughout], His118 and His196 are bonded to Zn1 (M1) with distances ranging 2.1–2.2 Å, and Asp120, His121, and His263 are for the second Zn2 (M2) with more variable distances, 3.0, 2.1, and 2.5 Å, respectively. The well‐ordered bridging water molecule is bonded to both zinc ions, with the distances of 2.5 Å to Zn1 and 2.1 Å to Zn2. Additionally, Zn1 contacts two oxygen atoms (2.2 and 2.4 Å) of an ethylene glycol, which was used as a cryo‐reagent, altogether in a hexadentate trigonal bipyramid configuration. For Zn2 (M2), the carboxyl group of Asp120 makes bifurcated, relatively longer contacts (3.0 and 3.3 Å) with the metal ion and additionally an ethylene glycol interacts (2.9 Å), which makes Zn2 site a hexadentate deviated from the tetrahedral configuration. The presence of the ethylene glycol, which contacts both zinc ions with two oxygen atoms, affected the di‐zinc active site, particularly the distance between the Asp120 and the zinc ions. In the other structures analyzed in this study and the previously reported (PDB IDs of http://firstglance.jmol.org/fg.htm?mol=5DPX, http://firstglance.jmol.org/fg.htm?mol=2FU8, http://firstglance.jmol.org/fg.htm?mol=5EVB, http://firstglance.jmol.org/fg.htm?mol=2FM6, http://firstglance.jmol.org/fg.htm?mol=2QDT, http://firstglance.jmol.org/fg.htm?mol=2AIO, and http://firstglance.jmol.org/fg.htm?mol=1SML) without ethylene glycol and with or without the other ligands (captopril, hydrolyzed antibiotics), the Zn─X (N or O) distances are maintained normal from 2.0 to 2.2 Å. Therefore, it depends on the crystallization conditions and the presence of ligands, the location and the distance of the bridging water (or hydroxide) molecule, and the zinc configuration change. See Table 1 for all Zn─X distances for L1 and L1 complexes.

**Table 1:** Zn‐X (N/O) distances in L1 structures

	L1‐Native	L1‐Capt	L1‐Mox	L1‐PenG	L1‐Mero	L1‐Imip	L1‐Mox‐Cd‐E	L1‐Mox‐Cu‐E
Zn1								
H116	2.1	2.1	2.1	2.1	2.1	2.2	2.3	2.2
H118	2.1	2.1	2.1	2.0	2.1	2.0	2.4	2.1
H196	2.0	2.0	2.1	2.0	2.0	2.2	2.3	2.2
bW	1.8	S‐2.2	1.9	1.8		1.9		
OC2			2.5	2.5	2.0		2.5	2.8
OH1						3.4		
Zn2								
D120	2.1	2.9	2.1	2.1	2.1	2.1	2.4	2.3
H121	2.0	2.0	2.1	2.0	2.1	2.2	2.4	2.1
H263	2.0	2.0	2.1	2.0	2.1	2.1	2.3	2.3
bW	2.0	S‐2.3	2.0	2.3		2.1		
N			2.5	2.3	2.0	2.2	2.5	2.6
OC1			2.3	2.3	2.4	2.4	2.5	2.3

Distances in Å, bW: bridging water, OC1: Carboxylate of 5‐ or 6‐membered ring, N: nitrogen in the 5‐ or 6‐membered ring, OC2: carboxylate/carbonyl originated from the β‐lactam ring, OH1: 1‐hydroxyethyl in Imipenem. “S”s in S‐2.2 and S‐2.3 indicate the sulfur atoms in the captopril molecules which replace the bridging water molecules

Several studies^5^, ^33^, ^34^, ^35^ indicated that affinities for Zn1 and Zn2 are in the nM to μM range (dissociation constants Kd) and quite different from each other in some cases. Particularly for B2 enzymes, as expected from being mono‐metal enzymes, the difference is as much as 1,000 folds.^35^, ^36^ Wommer and colleague reported that the dissociation constants for L1 are 2.6 and 6 nM for the zinc ions in Zn1 and Zn2 sites, respectively, showing slightly higher affinity for Zn1.^35^ In the L1 structures in this study, the differences in temperature factors and occupancies of the two zinc ions (or metal ions) are minimal, which correlates well with the reported biochemical studies.^5^, ^33^, ^34^, ^35^ However, although not included in this report, the EDTA‐treated structures with different EDTA soaking time where the crystals were soaked in less than 2 days have shown to have significantly different temperature factors and occupancies for the partially removed zinc ions perhaps indicating that these metal sites have distinct “off‐rates.”

**Figure 3:**
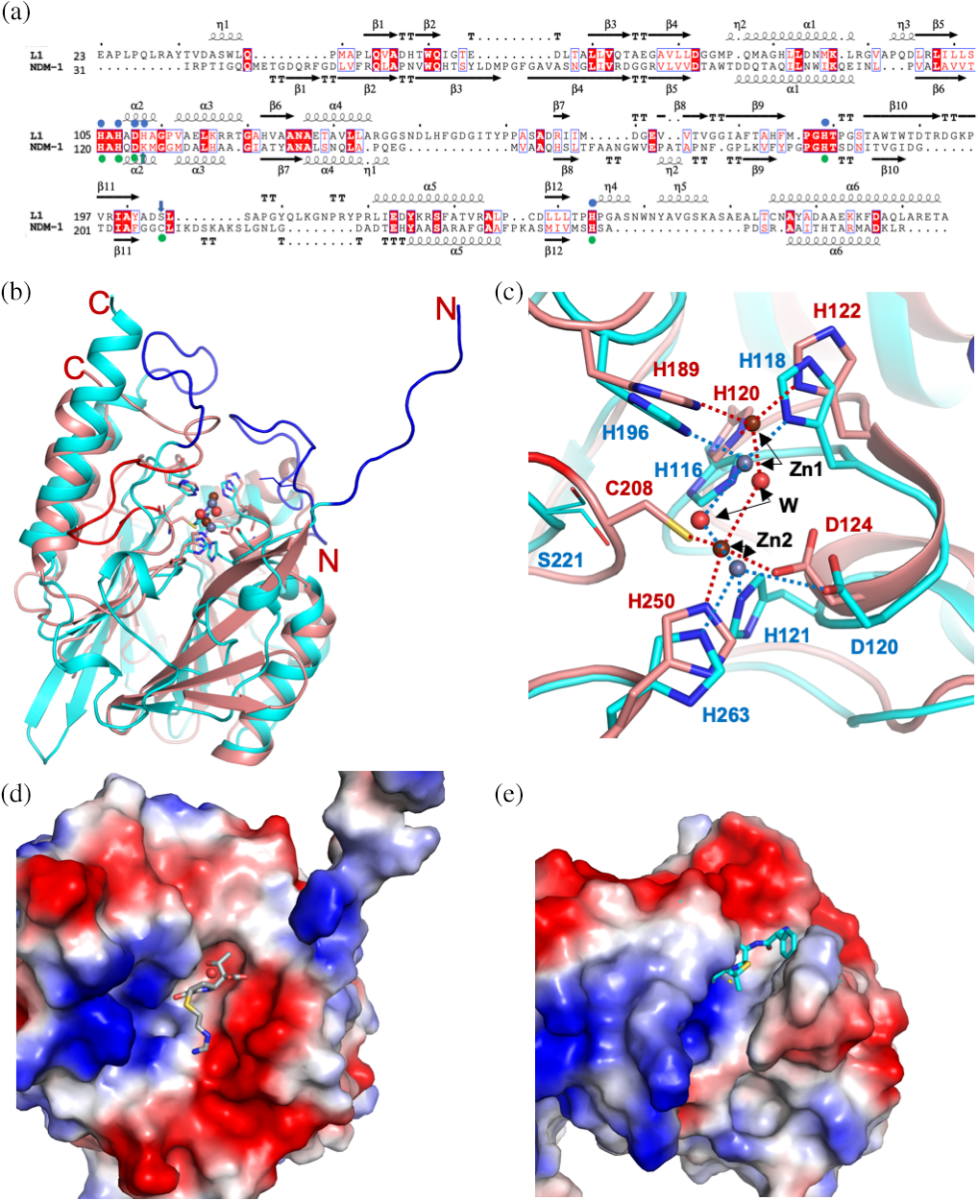
Comparison of the MBLs L1 and NDM‐1. (a) The structure‐based sequence alignment for L1 and NDM‐1, the zinc‐binding residues are shown with blue (L1) or green (NDM‐1) circles on top (L1) or bottom (NDM‐1) of the sequence. The residues of differences are indicated by arrows with the same protein colors, blue and green. For the structures (b and c), L1 is shown in cyan, and dark orange represents NDM‐1. Small spheres indicate zinc and ordered water molecules (red), dark red for NDM‐1 zinc atoms, gray for L1 zinc atoms. (C) Metal coordinating bonds are shown as a dashed line and colored red (for NDM‐1) and blue (L1). Zinc binding protein side chains are also depicted with colored sticks following the protein colors. The atomic color: blue for nitrogen, red for oxygen, carbon atoms follow protein colors. In (b) blue (L1) and red (NDM‐1) loops indicate the regions that are severely different from each other. The charge potential surface distribution of L1 (d) with bound hydrolyzed imipenem and NDM‐1 (e) with the ampicillin bound

When the active site of L1 is compared to that of NDM‐1 (see Figure 3a,c for conserved residues in the active sites), with Zn1, all histidine residues are conserved and the distances to Zn1 for NDM‐1 are in the range of 2.0–2.1 Å. At Zn2, Asp120 (Asp124 in NDM‐1) and His263 (His250 in NDM‐1) are conserved and the distances to Zn2 in NDM‐1 are 2.1 and 2.0 Å, which are quite similar to those of L1, except for the aforementioned native form that contains ethylene glycol in the active site. The third residue to contact Zn2 in L1 is His121 but that is replaced with Lys125 in NDM‐1 without contacting Zn2. Instead, Cys208 in NDM‐1 (Ser221 in L1) is replacing the role of His121 in L1 to interact directly with the zinc ion with a distance of 2.4 Å. Asp124 in NDM‐1 moves closer to Zn2 and the loop containing Cys208 is pulled in toward the zinc ion making the configuration somewhat different from that of L1. As a whole di‐zinc active site or di‐metal scaffold, the distance between the two zinc ions in L1 is 3.6–3.7 Å, which is much shorter than that of NDM‐1 (4.3–4.6 Å). The distances listed here for L1 are from the native form. However, for NDM‐1, these numbers are from the complex with hydrolyzed ampicillin. One important note here is that in NDM‐1, the distance between zinc ions depends on the pH of the crystallization conditions and varies between 3.5 Å at a high pH and 4.6 Å at a lower pH.^12^, ^37^ In all reported L1 structures, the crystallizations were done in narrow pH ranges of 6.5–8.0 and the resulting Zn–Zn distances fluctuate between 3.5 and 3.9 Å.

### 2.3 Comparison with NDM‐1

The L1 structures are compared to NDM‐1 structures, as this B1 MBL has been the most studied MBLs with more than hundred structures of NDM‐1 reported. The sequence identity between the two proteins is only 21% (see the structure‐based sequence alignment Figure 3a); however, overall folds are very similar and the RMSD of aligned and compared Cα atoms (179) is 2.2 Å, which is slightly larger than RMSDs for other B3 enzymes (<2.0 Å). There are a few considerably different regions as shown in Figure 3b. The N‐terminal 10 residue long loop in L1 is missing in NDM‐1 (NDM‐1 is a monomer) and the loop of Gly149‐Pro166 in L1 covering the substrate‐binding pocket at the top is absent in NDM‐1. The loop of Asp220‐Arg236 in L1 (blue) and Gly207‐Thr226 in NDM‐1 (red) adopts different conformations at the different locations relative to the di‐metal site. As shown in Figure 3b, the right side β‐sheet of L1 has shorter strands (cyan) than those of NDM‐1 (dark orange) suggesting an easier access of ligands (substrates) for L1. Due to these differences and the arrangement of the loops forming the binding pockets, the shapes of the pockets are different. Figure 3d,e displays the binding surfaces with similar charge potential distribution and the binding pockets of different sizes and shapes (i.e., more opened in different parts of the pockets). With these distinctive binding spaces, they may have different binding affinities to different groups of ligands toward the catalysis, which is reflected in the limited reported kinetic parameters Km (Michaelis–Menten constant) data shown in Table 1. L1 has lower Km values for penicillins (penicillin G and ampicillin) and some carbapenems but has higher Km values for other antibiotics than NDM‐1.^38^, ^39^, ^40^, ^41^

### 2.4 Comparison with other B3 enzymes

**Figure 4:**
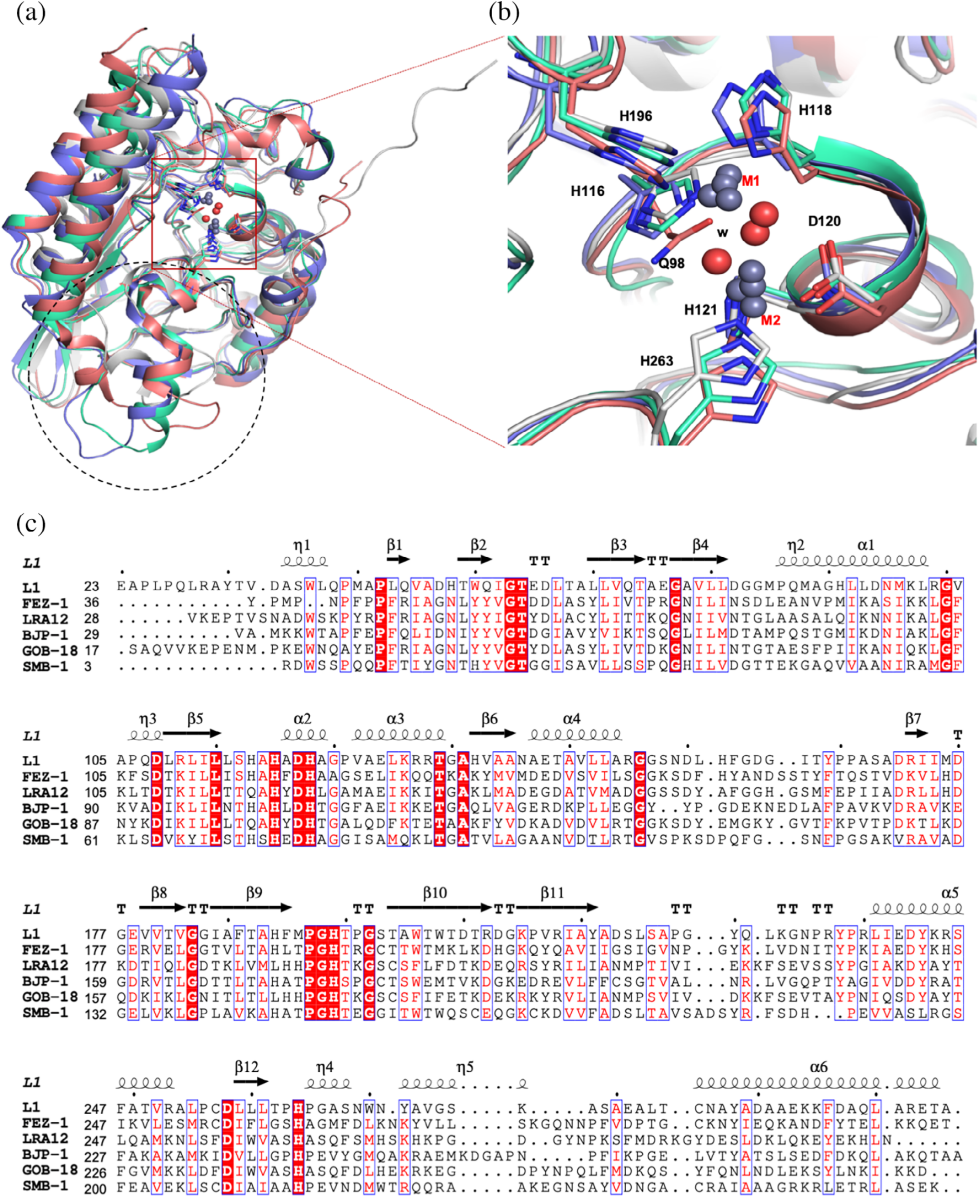
Comparison of MBL B3 enzymes. Four MBL B3 structures (only for clarity), L1 (gray), FEZ‐1 (cyan), BJP‐1 (purple), and GOB‐18 (pink), are superimposed. (a) The region in the black dotted circle exhibits a structurally variable region and the red square box shows the well‐conserved di‐nuclear zinc site. (b) The close‐up view of the di‐nuclear zinc sites, red square box in (a). GOB‐18 (pink) has Gln82 in place of H105 (L1). (c) The structure‐based sequence alignment of MBL B3 proteins

Except for the N‐terminal 10 residues tail in L1, which is shown to be a part of the dimer interface, the structures of B3 enzymes are very well conserved. When five B3 MBLs (FEZ‐1, LRA12, BJP‐1, GOB‐18, and SMB‐1) were compared to L1 (see Figure 4c for the structure‐based sequence alignment), the sequence identities were 23–33%, whereas the RMSDs of aligned Cα positions (226–242 a.a.) are 1.37–1.63 Å. There are some variations in the loops forming the binding site. Away from the binding site, residues Tyr271‐Thr289 in L1 of the long loop between β12 and α6 show a relatively big difference from other B3 proteins, which have a longer stretch containing one or two α‐helices (Lys270‐Cys290 in Fez‐1, Gln249‐Leu269 in BJP‐1, His248‐Tyr268 in GOB‐18) as shown in Figure 4a as the region in black circle. The di‐nuclear zinc sites, shown in the red square box in Figure 4a,b, are structurally well conserved except for GOB‐18, which has Gln82 in place of His116 (L1) still making a contact with Zn1 to maintain the di‐metal active site functional.

### 2.5 Structures of L1 complexes with hydrolyzed antibiotics

**Figure 5:**
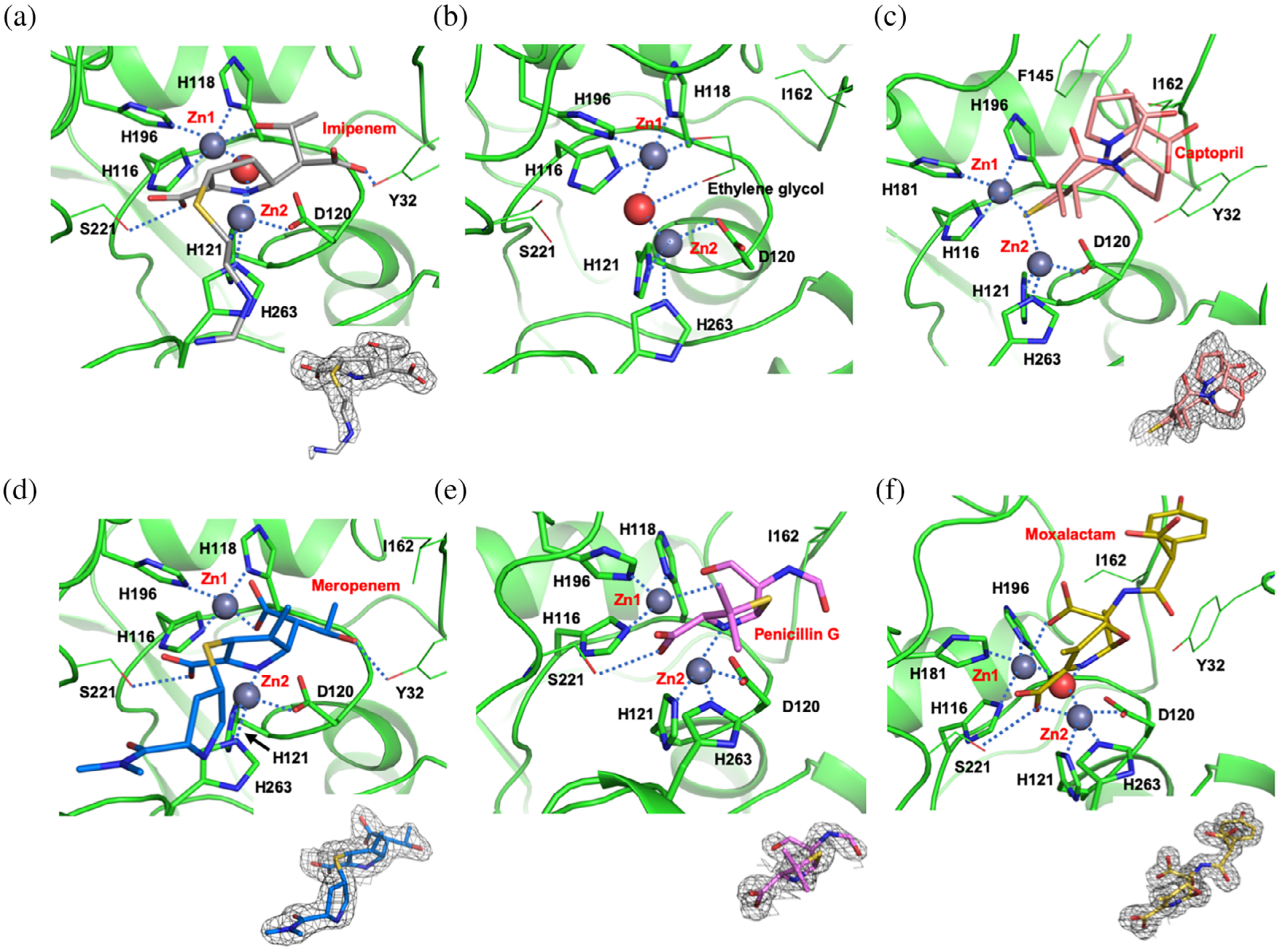
The active sites of the L1 structures. (a) L1‐imipenem, (b) native form, (c) L1‐captopril, (d) L1‐meropenem, and (e) L1‐penicillin G, (f) L1‐moxalactam. All antibiotics are in the hydrolyzed forms and depicted in sticks. The protein residues interacting with zinc ions are also depicted in sticks. The residues that interact with ligands are in line drawings. Electron density for the bound ligand is from the 2fo‐fc map, in the contour at 1.0 σ, except for the captopril, which is at 0.5 σ

More than 10 different antibiotics were tried for co‐crystallization or soaking and thus far, only penicillin G, meropenem, imipenem, and moxalactam were found hydrolyzed and bound in the active site. Presumably, these hydrolyzed antibiotics in the binding pocket may have higher affinities and be better inhibitor candidates than those not found in the trial structures. Careful analysis of the structures could lead to new or better inhibitors. When compared with the native form, all protein structures of the complexes are very similar to that of the native form including the position of active site side‐chain atoms. Overall, Cα RMSDs are less than 0.4 Å with 265 aligned and compared residues. None of the direct protein‐zinc (or other metals) interactions are changed or replaced in the complexes. In all L1‐hydrolyzed antibiotic complex structures, the amine/imine nitrogen of the 5‐ or 6‐membered ring interacts with Zn2 (or M2) and both oxygen of the carboxyl group that attached to the same ring interact with Zn2 (or M2) and OG atoms of Ser221 and Ser223. The carbonyl moiety attached to the lactam ring becomes a carboxyl or carbonyl group dependent on the local condition. Except for the L1‐Mero complex, the bridging water (or hydroxide) molecules are maintained in the same positions. There are weak long‐range hydrophobic interactions between hydrolyzed antibiotics and protein residues in the pocket such as Trp39, Phe156, Ile162, and Pro226. Some of the extended parts of lactam antibiotics, away from the opened lactam ring, which contacts a metal directly, are disordered and not well‐defined (Figure 5). See Table 1 for all Zn─X distances in the L1 and L1 complexes.

*L1‐PenG (penicillin)* (Figure 5e): Overall, the structure of the hydrolyzed penicillin is not defined well. The carbonyl oxygen, a part of the opened lactam ring, but not carboxylated, interacts with Zn1 (2.5 Å), similarly to other carboxylated hydrolyzed β‐lactam antibiotics mentioned above. The other carbonyl oxygen in the phenylacetamido group attached at position 6 of the original lactam contacts OH of Tyr32 residing at the C‐terminal end of the N‐terminal arm (residues 23–32), which is a main part of the dimer interface. The phenyl group is disordered and not visible. The other hydrolyzed penicillin G structures in the NDM‐1‐hydrolyzed penicillin G complexes (PDB IDs http://firstglance.jmol.org/fg.htm?mol=4RAM and http://firstglance.jmol.org/fg.htm?mol=4EYF) are better defined. In the NDM‐1 structure, Trp93 and Met63 provide hydrophobic interactions for the phenyl group of penicillins. However, the phenyl group of penicillin G in L1‐PenG, if it were to follow a similar orientation and location to that in the NDM‐1 complex, can be in position to clash with the side chain of Tyr32. Instead, it orients upward and gets exposed to solvent, which may lead to disordering. Although NDM‐1 has higher Km values (~200–300 μM) for penicillins (penicillin G and ampicillin) than L1 (~40–50 μM) (see Table 2), the hydrolyzed penicillins can be more stable in the NDM‐1 binding pocket than in the L1 binding pocket.

**Table 2:** Catalysis/affinity (Km) comparison of NDM‐1 and L1^38^, ^39^, ^40^, ^41^, ^42^, ^43^

Km	NDM‐1 (μM)	L1 (μM)	References
Penicillin G	240	55	38, 39, 40, 41
Ampicillin	310	40	38, 41
Imipenem	45	90	38, 40
Meropenem	54	10	38, 39, 40
Moxalactam	54	1	41, 42
Nitrocefin	1	7	38, 41
Cefepime	77	>1,000	39, 40
Cefotaxime	10	26	39, 41
L‐captopril	4	5	43

*L1‐Imip and L1‐Mero (Carbapenems)* (Figure 5a,d, respectively]: In addition to the opened lactam ring interaction with zinc ions, one oxygen of the carboxyl group converted from the carbonyl of the lactam ring after hydrolysis of meropenem replaces the bridging water and makes bifurcated contacts to both zinc ions (2.0 Å to Zn1 and 2.8 Å to Zn2,). The hydroxyl oxygen of the 1‐hydroxyethyl group at position 6 of the carbapenem contacts OH of Tyr32 (Figure 5d). All reported structures (PDB IDs http://firstglance.jmol.org/fg.htm?mol=5N0H, http://firstglance.jmol.org/fg.htm?mol=4YEL, http://firstglance.jmol.org/fg.htm?mol=4RBS, http://firstglance.jmol.org/fg.htm?mol=5YPM, and http://firstglance.jmol.org/fg.htm?mol=5YPN) of the hydrolyzed meropenem (hydrolyzed by L1 and NDM‐1) have similar conformations with the carboxyl oxygen replacing the bridging water molecules. One exception is in the VIM‐1‐hydrolyzed meropenem structure (PDB ID http://firstglance.jmol.org/fg.htm?mol=5N5I), where the carboxyl group rotates about 60° counter clockwise (CCW) along the C5–C6 (see the carbapenem numbering in Figure 1) and does not interact with zinc ions so the bridging water maintains its position. However, in the hydrolyzed imipenem structure, the same carboxyl group and the 1‐hydroxyethyl group rotate about 180° along the C5–C6 switching their positions, the hydroxyl oxygen contacts Zn1 and the carboxyl oxygen interacts with OH of Tyr32. The bridging water molecule keeps the position. The structure of the hydrolyzed imipenem by SMB‐1 (PDB ID http://firstglance.jmol.org/fg.htm?mol=5B1U) has the same conformation. However, other hydrolyzed imipenem (PDB IDs http://firstglance.jmol.org/fg.htm?mol=5YPI, http://firstglance.jmol.org/fg.htm?mol=5YPL, and http://firstglance.jmol.org/fg.htm?mol=5YPK), structures with NDM‐1 adopted the conformation of the hydrolyzed meropenem. Both pyrrolines of hydrolyzed imipenem and meropenem seem to adopt a similar Δ1β tautomer^44^, ^45^ as the double bonds shift from C2‐C3 to C3‐N4 as evidenced by puckering of the pyrroline rings—the sulfanyl sulfur at position 2 is not in the C1‐C2‐C3 plane. This suggests that in the later catalytic steps, the hydrolytic intermediates are protonated from bulk solvent water molecule on the same side of the bridging water molecule and Zn1. This tautomerization is different from that for hydrolyzed moxalactam, which adopts a pyrroline tautomer close to Δ2, implying a different catalytic step. The long linear sulfanyl substituent ([aminomethylideneamino]ethylsulfanyl) in the pyrroline ring of the imipenem is not well contacted by protein atoms and only partially defined (Figure 5a). In the hydrolyzed meropenem, however, the bulkier thio‐substituent of the pyrrolidine ring is in the neighborhood of His263 and Trp38 and is better defined. This indicates that the shape and size of the ligand matter to stay stable in this pocket.

*L1‐Mox (Cephalosporin)* (Figure 5f): The L1‐Mox structure, both the protein and the ligand, is very similar to that previously reported (PDB ID http://firstglance.jmol.org/fg.htm?mol=2AIO),^24^ with the RMSD of 266 compared protein Cα atoms of 0.32 Å and the average deviation of less than 0.2 Å for the hydrolyzed moxalactam. To briefly describe, with two zinc ions, one of the carboxyl oxygen from the lactam ring directly interacts with Zn1 and the nitrogen atom, and a carboxyl oxygen in the six‐membered oxazine ring contacts Zn2. The oxygen in the methoxy substituent and the carbonyl oxygen in the hydroxyphenylacetylamino substituent interact with Tyr32 oxygen (OH), and two carboxyl oxygen in the six‐membered oxazine ring contact OG atoms of Ser221 and Ser223, thus stabilizing the compound in the binding pocket.^24^ As in the case for the Spencer's structure,^24^ the tetrazole‐containing thiomethyl substituent at position 3 in the oxazine was either completely disordered or hydrolyzed by another mechanism and not visible. The hydrolyzed moxalactam is the best‐defined molecule among all hydrolyzed antibiotics bound to L1 in this study. Interestingly, the structure of the L1‐moxalactam has the largest Cα RMSD with 0.76 Å suggesting that the protein makes slightly larger changes (than those with other antibiotics) to interact with the intrinsically better‐fitted molecule.

### 2.6 Structure of L1‐captopril

D‐ or L‐captopril has been one of the most popular starting thiol‐based inhibitors for metalloenzymes as it can form a specific ternary complex enzyme‐metal‐inhibitor, which provides better selectivity toward drug targets than those of metal‐stripping type inhibitors (Figure 5c). In the structure of the complex, the captopril molecule adopts two conformations and both are not well occupied (occupancy of ~0.5 or less). There is another not well‐defined captopril molecule found between the two protein molecules. Both conformers of the captopril interact with both zinc ions using a sulfur atom, which replaces the bridging water molecule. As the two conformers are 180° away from each other along the C2─C3 bond, the carbonyl oxygen face the exact opposite direction, one of them interacting with OH of Tyr32 and both proline parts (with carboxyl group) extended to the protein surface. Except for the cysteine moiety including the sulfur atom, which contacts both zinc ions, the L‐captopril (or D‐captopril) structures in other MBL‐captopril complexes (PDB IDs of http://firstglance.jmol.org/fg.htm?mol=2FU8, http://firstglance.jmol.org/fg.htm?mol=5ZJ2, http://firstglance.jmol.org/fg.htm?mol=4EXS, and http://firstglance.jmol.org/fg.htm?mol=4C1C)^17^, ^46^ have the different conformation, placing the proline moiety and the carbonyl group in location in the pocket different from the two alternative conformations of L‐captopril in the L1‐Capt structure by the rotation of 120° along the C2─C3 bond. Dependent on L‐ or D‐ configuration of the proline moiety, the carboxyl group is facing a different side of the binding pocket and in the L1‐D‐captopril complex (2FU8),^17^ the carboxyl oxygen makes a hydrogen bond to OG of Ser223. The binding site of L1 (or other MLB) can accommodate various forms of a captopril molecule suggesting utilizable room to introduce derivatives for higher selectivity and affinity.^20^, ^47^ The L1 structures are quite similar and all inter‐metal distances in the structures are around 3.7–3.8 Å.

### 2.7 Structures of metal‐free L1

**Figure 6:**
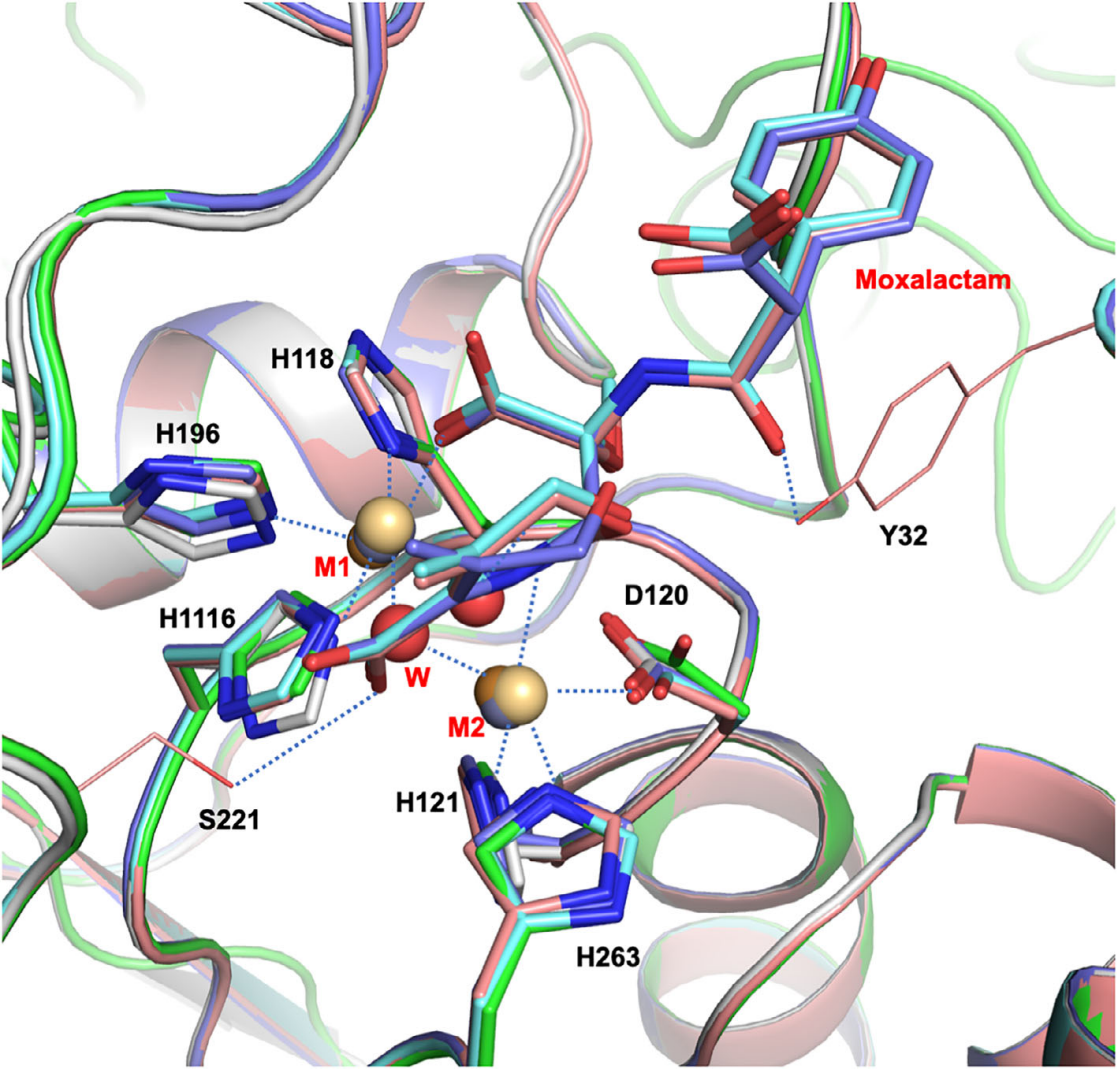
The structures from EDTA‐treated crystals, L1‐E, L1‐E‐Cu‐MT, and L1‐E‐Cd‐MT, are shown to be very close to those of untreated structures with RMSD values for Cα atoms of less than 0.4 Å. L1‐Native (green), L1‐E (gray), L1‐E‐Cd‐MT (cyan), L1‐E‐Cu‐MT (dark blue), L1‐Mox (dark orange), M1 and M2: are Zn1 and Zn2 (gray), Cd1 and Cd2 (pale orange), Cu1 and Cu2 (dark orange)

Wommer^35^ suggested that MBL might be in a metal‐free form in vivo until it binds β‐lactam antibiotic, induces a change in the active site conformation, and then binds metal. To better understand the interactions of metals with the enzyme and its ligands, the structures of L1 were determined in the absence and presence of different metals with the hydrolyzed moxalactam. The crystals of metal‐free enzyme were prepared by soaking with EDTA. The protein structure was maintained during the soaking with EDTA for 2 days and the subsequent washing. The crystal diffracted to 1.90 Å with the slightly elevated B‐factors (26.2 vs. 35.5 Å^2^). The active site conformation was quite similar to that of the structures with the metals and with bound ligands (Figure 6). Zn1 was replaced by a water molecule, no other molecule was identified in the Zn2 position and the bridging water was not found. More precisely, the distances between the water molecule, which replaced Zn1 and the nitrogen atoms in the histidine residues, are lengthened by 0.1–0.6 Å suggesting the possibility of the presence of a trace amount of zinc ion. The protein main chain atoms did not move much at all (RMSD for Cα atoms is 0.35 Å). In the active site, the side chain of His118 did not move, the side chains of His196 and His116 turned about a degree toward the water molecule, and Asp120 was drawn close to the water by about 0.2 Å. Although not reported here, we determined the crystal structures from the crystals soaked with EDTA for 1 and 2 hr. These structures contain both zinc ions but with noticeably lower and two different occupancies. Apparently, two zinc ions can be removed without breaking the crystal lattice implicating that zinc binding does not require significant conformational changes. It also indicates that Zn1 is the higher affinity site or it has a lower off‐rate.

Interestingly, when the antibiotics were soaked into the EDTA‐treated crystals without metal ions, no antibiotic molecule was found in the structure. However, when the metal, cadmium or copper was soaked back in, followed by the antibiotics, the hydrolyzed antibiotics were found in the binding site. This suggests that metal ions are required for substrate binding. All structures of the complexes from the EDTA‐treated crystals are virtually the same as those from non‐treated crystals including the interactions between the metal‐protein residues and inter‐metal distances (Figure 6). The structure of L1‐Mox‐Cu‐E was compared with the structure of the Cu^2+^ bound form of L1 by the Dideberg group.^17^ As expected, these two L1 structures are quite similar to an RMSD of 0.32 Å with 266 Cα atoms. In the active site, the average difference of the matching metal–ligand distances is 0.16 Å longer for the structure of L1‐Mox‐Cu‐E. The positional differences between matching metal atoms, Cu1‐Cu1′ and Cu2‐Cu2′ are 0.15 and 0.27 Å, respectively. Interestingly, two ordered water molecules, one is binding to Cu2, and the other one is hydrogen bonding to OG of Ser223, are replaced with carboxyl oxygen of hydrolyzed moxalactam in the L1‐Mox‐Cu‐E structure.

The structure analysis of L1 with different metals revealed that all inter‐metal distances, Zn–Zn, Cd–Cd, and Cu–Cu are very similar regardless of the presence of ligand with the distance of 3.6–3.7 Å, which is strikingly different from those of NDM‐1. NDM‐1 has a longer metal–metal distance for zinc ions with 4.6 Å, but shorter with cadmium ions. To be more precise, the Cd1 site is split into two in both protein chains in the asymmetric unit in the structure of the NDM‐1 with cadmium. The highly occupied Cd1 is located closer to Zn1 in the NDM‐1 structure with zinc ions, and the Cd1‐Cd2 distance is 4.3 Å, whereas the second Cd1 site has a lower occupancy and has a much shorter Cd1‐Cd2 distance of 3.7 Å. This is notably important because NDM‐1 in the presence of cadmium ions has shown to have a lower activity and the intermediate structure could be captured by the crystal structure.^12^ Other related MBLs,^25^, ^27^, ^34^, ^46^ VIM‐2 (subclass B1), IMP‐1 (B1), FEZ‐1 (B3), and BJP‐1 (B3), have the distances (3.4–3.8 Å) similar to that of L1 than that of the NDM‐1 with zinc ions.

### 2.8 Biochemical analysis of metal dependency of L1 with stability and activity

The roles of the di‐metal scaffold in stability, kinetic property, and enzyme activity for L1 were investigated by differential scanning fluorimetry (DSF), isothermal titration calorimetry (ITC), and enzyme assays in comparison with NDM‐1.

#### 2.8.1 Differential scanning fluorimetry

**Figure 7:**
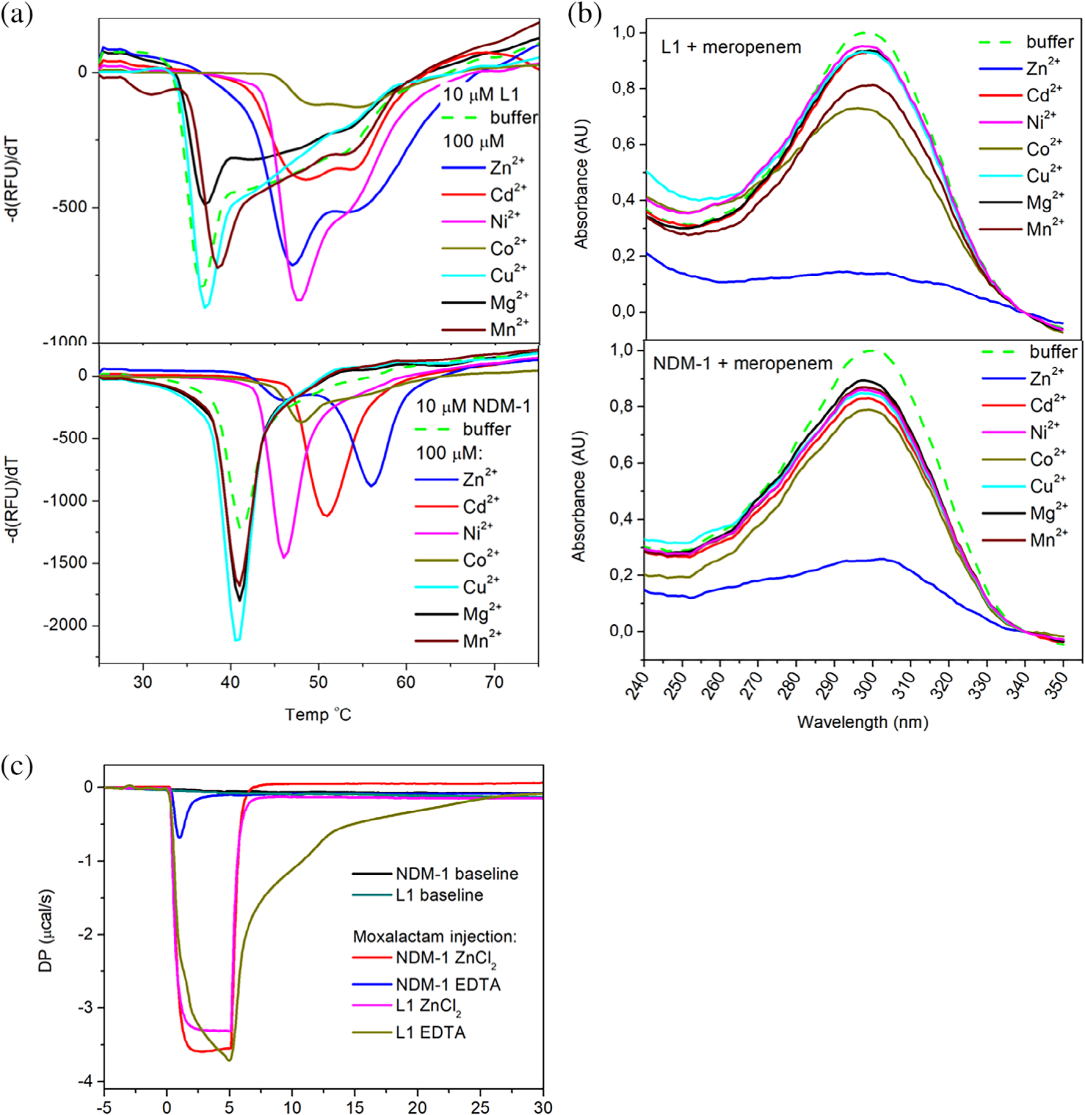
Biochemical analysis of L1 and NDM‐1 with and without metal ions. (a) Melting temperature analysis of L1 and NDM‐1 by DSF. (b) Metal‐dependent enzymatic activity analysis of L1 and NDM‐1 with meropenem. (c) Binding kinetic analysis of moxalactam by ITC

Among metal(s) required for MBLs for their substrate binding and optimum catalytic activity, the natural metal that appears most is zinc. However, several metals were reported to substitute zinc with variable catalytic activity. For example, cadmium ions have shown to have significantly reduced activity for NDM‐1.^12^ Metal ions in metalloenzymes are directly involved in the catalytic activity. Moreover, often they contribute to stabilizing metalloenzymes, which is closely related to their catalytic activities. To investigate the role of metal ions in the stability of L1 and NDM‐1, DSF was used to examine thermal stability of the proteins in the presence of several metals including Zn^2+^, Cd^2+^, Cu^2+^, Co^2+^, Ni^2+^ and Mn^2+^, as well as Mg^2+^, as a control (see Figure 7a). As expected, Mg^2+^ did not contribute to the stability of both MBLs. Without metals, NDM‐1 is slightly more thermally stable than L1 as they melt at 41 and 37°C, respectively. In the presence of an excess amount (20‐fold excess of the protein concentration) of Zn^2+^, NDM‐1 melts at 15°C higher indicating that the enzyme is much more thermally stable. The melting curve indicates a very small additional melting point at 46°C, which suggests that one zinc ion might leave at the temperature but the protein may still maintain its integrity. Other metals showed the different degrees of melting point shifts but all showed lower melting temperature (Tm) than zinc and each curve indicates only one melting point. It is shown larger shifts in the order of Zn (15°C) > Cd (10°C) > Co (7°C) > Ni (5°C) (see Table 4 for detailed temperature shift). Copper and manganese did not affect the Tm at concentrations up to 100 μM (protein concentration is at 10 μM). If the stability of the enzyme was to correlate with its catalytic activity, NDM‐1 may maintain activity even after losing the first zinc ion. However, with other metals, as the enzyme loses both metals at once, it may lose its activity quickly as well. For L1, all melting curves are bimodal, with or without metal, reflecting a two‐step thermal denaturation, which perhaps includes the breakdown of tetrameric assembly. The mutational study reported that the formation of the tetramer is critical for the catalytic activity and/or substrate binding profile.^31^ Another possibility could be the presence of the disulfide bond between the residues Cys256 and Cys290. However, this disulfide bond is found only in the structures of the complexes between L1 and meropenem, penicillin G or moxalactam with copper ions suggesting that this disulfide bond may not contribute significantly to stability and/or catalytic activity. It is the second Tm at 37°C that is close to the monomeric NDM‐1's Tm (41°C) without a metal ion. Both zinc and nickel show a similar significantly elevated Tm (~55°C) and cadmium and cobalt also share a similar high Tm. Manganese and copper again did not affect Tm of L1 at the same concentration. Both proteins have shown that metals, particularly zinc ions, provide significant stability. However, they have different profiles with different metals: with NDM‐1, different metals give distinct stability, but L1 seems to maintain similar stability with different metals, perhaps having to contain a di‐metal configuration that is subtly different. This DSF analysis suggests that with distinct di‐metal active site architectures, NDM‐1 from subclass B1 and subclass B3 enzyme L1 may produce diverse catalytic activity by adapting differently in a given environment with different metal contents.

#### 2.8.2 Enzymatic activity analysis

Our DSF analysis has shown that different metals (thermally) stabilize the enzymes differently. Enzymatic activities are observed for the same metals used for DSF analysis to learn how well these metals replace zinc ions. The metal‐dependent enzymatic activity of L1 and NDM‐1 is characterized by one of the carbapenems, meropenem, which has a characteristic absorbance near 300 nm (Figure 7c). In the presence of the canonical metal zinc, which was added after EDTA‐treatment, both L1 and NDM‐1, as expected, hydrolyzed most of the meropenem after a 1‐min incubation (Figure 7c). With all other metal ions, only a minimal degree of hydrolysis was made for NDM‐1. For L1, except for Co^2+^ and Mn^2+^, a similar progression is observed. It is known that some metal ions can replace zinc ions with a comparable catalytic efficiency. Perhaps, when zinc ions are depleted by EDTA, the protein may undergo some conformational changes, albeit subtle, and it may take some time to recover, presumably in a metal‐specific manner, even with an excess amount of metal ions. After EDTA‐treatment, both proteins may have the conformation in which the canonical metal zinc can bind without much change (conformation and energy). Thus, the recovery with zinc was fast but not with other metals.

#### 2.8.3 Kinetic profile analysis using isothermal titration calorimetry

The structural information from the EDTA‐treated crystals indicated that metal ions are required for specific ligand binding. To better understand the dynamic properties of ligand binding by L1 and NDM‐1 in comparison, the enzymes treated with EDTA to remove most of the zinc ions in the protein were titrated with moxalactam using ITC. NDM‐1 and L1 also show the distinct kinetic profiles in the presence and absence of zinc ions as revealed in the ITC analysis^48^ (Figure 7b). The binding of antibiotics to MBL is an exothermic reaction as shown in the ITC experiment of L1 or NDM‐1 being titrated with a single slow injection of the antibiotic substrate moxalactam. In the presence of zinc ions, both enzymes process the substrate very quickly and efficiently as soon as being injected, and the product, hydrolyzed moxalactam, with low affinity leaves the binding pocket without affecting the heat profile (ΔP:μcal/s). For this reason, the binding pocket may not be filled to reach equilibrium. It implicates that in the presence of zinc ions, the substrate may have a similar on‐rate to both L1 and NDM‐1 and the product (hydrolyzed moxalactam) may have a similar off‐rate, but higher than that for the substrate, to both enzymes. Once the substrate injection completes, hydrolysis also finishes, and it reaches equilibrium. In the absence or limited amount of zinc, L1 and NDM‐1 have very different activities. Although low affinity, moxalactam having a higher on‐rate than off‐rate with NDM‐1 would fill the binding site and reaches the equilibrium quickly. However, for L1, substrates, with a comparable on‐rate and off‐rate, which may be lower than those with NDM‐1, actively bind and get released until the substrate injection finishes. Since the off‐rate of the substrate is low, they dissociate slowly, and the system goes to the equilibrium very slowly. This experiment also shows diversity in substrate binding in the presence and absence of zinc, which implicates a disparate catalytic activity for different MBL family proteins.

## 3 CONCLUSIONS

In this study, we report structural, thermodynamic, and enzymatic analyses of the class B3 L1 MBL from *S. maltophilia* and compare it to properties of class B1 NDM‐1 MBL.

The structures of L1 in complexes with hydrolyzed antibiotics and the inhibitor captopril with zinc or other metals showed that there is no significant structural difference between the native form and the complexes with ligands and with different metals. Most of the direct contacts made by the ligands are to two Zn^+2^ ions or the other metal ions that replace zinc. Only a few residues of the protein, Tyr32, Ser221, and Ser223, are involved in the direct interaction with ligands. The protein provides a large binding space to accommodate a broad range of β‐lactam antibiotics, which bind directly to the di‐metal scaffold formed at the bottom of the pocket in the enzyme. This di‐metal scaffold with Zn^+2^ ions (or other metals) mainly carries out the catalysis and no protein atom other than side chains forming the di‐metal scaffold participates directly in the catalytic reaction.

The L1‐structure without Zn^+2^ ions was produced by treating crystals with EDTA. Analysis of the structures from the EDTA‐treated crystals, apo form, complexes with moxalactam, and different metals has shown that all structures maintained much of the same conformation as those of structures from untreated crystals. The L1 structures from EDTA‐treated crystals, soaked in moxalactam solution without any metal, did not contain a ligand, thus supporting the notion previously suggested by the biochemical study for B3 MBL GOB‐18 that the functional metal scaffold is required not only for catalysis but also for β‐lactam binding.^11^, ^49^

Biochemical analysis by DSF assay has shown that metal ions increase stability to both L1 and NDM‐1. However, NDM‐1 and L1 have different stability profiles with different metals. Zinc is most favorable for NDM‐1's stability and other metals provide varying degrees of stability. For L1, many metals including zinc provide similarly high degrees of stability. Kinetic analysis by ITC^50^ revealed that L1 and NDM‐1 seem to have different substrate and product affinities and different on‐ and off‐rates in the presence and absence of metal.

Our study suggests that the metal scaffold is central to MBL's functional activity, contributing to stability, substrate binding, and catalysis. L1 of subclass B3 MBL and the B1 MBL enzyme NDM‐1 have similar but distinct metal scaffolds, which contribute to differences in substrate binding and catalysis by the MBL.

Our study implicates that MBLs of different subclasses have subtly different di‐metal scaffolds, which may confer diversity in substrate binding and catalysis.^39^, ^51^ This diversity may be crucial in adapting to a given environment in a manner relevant to the development of resistance. This information can be used to guide lead optimization for developing better drugs against MBLs, which are considered environmentally critical.

## 4 MATERIALS AND METHODS

### 4.1 Cloning of expression plasmids

The gene cloning was performed according to Kim et al.^52^ L1 (residue 20‐290) from *S. maltophilia K279a* was amplified from the genomic DNA with KOD Hot Start DNA polymerase. For the construct, 5′ TACTTCCAATCCAATGCCAGCGCCGCCGAGGCAC forward and 5′ TTATCCACTTCCAATGTTAGCGGGTCCCGGCCGTTT reverse primers were used. The PCR products were purified and cloned into the pMCSG53 according to the ligation‐independent procedure^53^, ^54^ and transformed into the *E. coli* BL21(DE3)‐Gold strain (Stratagene). A single colony was picked, grown, and induced with isopropyl‐β‐d‐thiogalactoside (IPTG). The cell lysate was analyzed for the presence of the protein with the correct molecular weight. The solubility was analyzed via small‐scale Ni‐NTA affinity purification and overnight tobacco etching virus (TEV) protease cleavage followed by centrifugation at 3,200*g* of digested sample. For large‐scale purification, a fresh transformation was made into the *E. coli* BL21‐Gold (DE3) strain.

### 4.2 Protein expression and purification

For purification of the protein used for crystallization, a 1 L culture of enriched LB medium was grown at 37°C (190 rpm). At OD_600_ ~1.0, the culture was cooled down to 4°C and 10 ml of 4 M K_2_HPO_4_ was added. Protein expression was induced overnight at 18°C with 0.5 mM IPTG. Bacterial cells were harvested by centrifugation at 7,000*g* (Sorval evolution RC centrifuge, Thermo Scientific) and cell pellets were resuspended in a 35 ml lysis buffer (500 mM NaCl, 5% [v/v] glycerol, 50 mM HEPES pH 8.0, 20 mM imidazole, and 10 mM β‐mercaptoethanol) with protease inhibitor cocktail (Complete Ultra‐EDTA‐free, Sigma) per liter culture and treated with lysozyme (1 mg/ml) and sonication. Debris was removed by centrifugation. L1 protein was purified via Ni affinity chromatography using the AKTAxpress system (GE Health Systems). The column was washed with 20 mM imidazole (lysis buffer) and eluted in the same buffer containing 250 mM imidazole. Immediately after purification, the His‐tag was cleaved at 4°C for 20 hr using a recombinant His‐tagged TEV protease, resulting in an untagged protein with an N‐terminal Ser‐Asn‐Ala peptide. An additional Ni affinity chromatography purification was performed to remove the protease, uncut protein, and affinity tag. The purified protein was then dialyzed against a 15 mM HEPES pH 7.0, 100 mM NaCl, and 2 mM TCEP buffer.

The protein concentration was estimated by UV absorption spectroscopy (280 nm) using a NanoDrop 1000 spectrophotometer (Thermo Scientific). The purified L1 was concentrated using a centrifugal filter (10 kDa MWCO, Amicon‐Millipore) to 71 mg/ml.

### 4.3 Crystallization

Initial crystallization screening was done with the sitting‐drop, vapor‐diffusion technique in 96‐well CrystalQuick plates (Greiner Bio‐one) using a Mosquito robot (TTP LabTech) and commercial crystallization kits including MCSG 1‐4 (Anatrace), Index (Hampton Research), and Peg‐ion (Hampton Research). The protein concentration used was 15 mg/ml. The L1 protein construct used for this study crystallizes readily in several different conditions in the presence of zinc ions. Among many diffraction quality crystals, the best crystals of the native form (L1‐Native) were monoclinic P2_1_, which appeared within a week in condition #77 (G5) of the MSCG‐4 containing 0.1 M HEPES pH 7.5, 10% (w/v) PEG 4000. Since no ligands would co‐crystallize, all crystals of the complexes were prepared by soaking the native form crystals in the same crystallization solution with the ligands. For example, L1‐PenG crystals were prepared from the crystals grown in the condition Index A4 (0.1 M Bis‐Tris pH 6.5, 2.0 M ammonium sulfate) by being soaked in the same solution plus 5 mM penicillin G. Similarly, L1‐Capt crystals were prepared from the Peg‐Ion condition F4 (8% v/v Tacsimate, pH 7.0, 20% w/v PEG3350) with 25 mM captopril. L1‐Imip crystals were produced from the Peg‐Ion condition E8 (0.2 M sodium malonate, 20% w/v PEG 3350) with 5 mM imipenem, L1‐Mero crystals were prepared from the Index condition H2 (0.2 M tartrate, 20% w/v PEG3350) with 5 mM meropenem, and L1‐Mox crystals were from soaking in Index H10 (0.2 M citrate, 20% w/v PEG3350) with 5 mM moxalactam. Prior to flash freezing in liquid nitrogen, crystals were cryo‐protected by briefly soaking in the crystallization solution containing up to 25% ethylene glycol.

Crystals of L1‐E, L1‐Cd‐Mox‐E, and L1‐Cu‐Mox‐E were prepared from the crystals produced in the condition PEG‐Ion E8. For L1‐E, the crystals were soaked in the same well solution additionally containing 5 mM EDTA for up to 48 hr. After given times, crystals were washed briefly (30–45 s) three times in the cryo‐solution, which contains an additional 25% ethylene glycol to remove most of the EDTA on the crystals, checked for diffraction, and used for data collection and structure determination. For L1‐Cd‐Mox‐E and L1‐Cu‐Mox‐E, the crystals were soaked for 48 hr with 5 mM EDTA and then washed with the same well solution (without EDTA) three times over 5 min, then (a) soaked in the solution containing 10 mM moxalactam and checked for diffraction or (b) soaked in 10 mM CdCl_2_ followed by another soaking with 10 mM moxalactam (L1‐Cd‐Mox‐E), or (c) soaked in CuCl_2_ followed by another soaking with 20 mM moxalactam (L1‐Cu‐Mox‐E). All soakings were done for 10 min.

### 4.4 Crystallographic data collection and data processing

The crystal diffractions were measured at a temperature of 100 K using either a 0.5 s exposure per 0.5° rotation or a 1 s exposure per 1° rotation on *ω* for 90–305°. Data for the crystals of L1‐native (up to 1.70 Å resolution) and L1‐Capt (1.40 Å) were obtained at SBC‐19ID beamline on the dectris Pilatus3X 6M detector, and those of L1‐Mox (1.52 Å), L1‐PenG (1.65 Å), L1‐Imip (1.90 Å), L1‐Mero (1.98 Å) were collected at SBC‐19BM. They were recorded on the ADSC Q210r detector both with an X‐ray wavelength near 12.66 keV (0.97927 Å) for molecular replacement (MR) phasing (SBC, Advanced Photon Source, Argonne, Illinois). Similarly, the data for EDTA‐soaked crystals (called L1‐E) and for the crystals of the EDTA‐soaked then treated with additional metal and antibiotic soaking (called L1‐Cd‐Mox‐E and L1‐Cu‐Mox‐E) were collected. All diffraction images were processed using the HKL 3000 suite^55^ (see Table 3).

**Table 3:** Data collection and refinement statistics

	L1‐Native	L1‐Capt	L1‐Mox	L1‐PenG	L1‐Mero	L1‐Imip	L1‐E	L1‐Mox‐Cd‐E	L1‐Mox‐Cu‐E
Wavelength, Å	0.97918	0.979226	0.97919	0.91916	0.97919	0.97935	0.97911	0.97911	0.97911
X‐ray source	SBC‐19ID	SBC‐19ID	SBC‐19BM	SBC‐19BM	SBC‐19BM	SBC‐19BM	SBC‐19BM	SBC‐19BM	SBC‐19BM
Resolution limit, Å	48.6–1.70 (1.73–1.70)	46.2–1.40 (1.42–1.40)	35.8–1.52 (1.55–1.52)	34.3–1.65 (1.68–1.65)	35.9–1.98 (2.01–1.98)	34.3–1.90 (1.93–1.90)	41.2–1.80(1.83–1.80)	43.4–1.60 (1.63–1.60)	46.1–2.38 (2.42–2.38)
Space group	P2_1_	P6_4_22	P6_4_22	P6_4_22	P6_4_22	P6_4_22	P6_4_22	P6_4_22	P6_4_22
Unit cell (*a, b, c, α, β, γ*, Å, °)	68.03, 97.23, 91.73, *β* = 105.6	*a* = 104.53, *c* = 98.72	*a* = 103.92, *c* = 98.93	*a* = 104.76, *c* = 98.43	*a* = *b* = 104.41, *c* = 98.85	*a* = *b* = 104.84, *c* = 98.63	*a* = *b* = 104.47, *c* = 99.04	*a* = *b* = 104.34, *c* = 98.98	*a* = *b* = 104.23, *c* = 98.79
# molecules in ASU	4	1	1	1	1	1	1	1	1
Unique reflections	122,234 (6033)	63,060 (3071)	48,271 (1818)	38,907 (1892)	22,788 (1101)	26,020 (1258)	29,676 (1215)	42,294 (1952)	13,193 (638)
Multiplicity	5.9 (5.8)	9.6 (4.8)	17.3/5.3)	20.8 (16.0)	20.5 (17.6)	20.2 (20.4)	14.6 (6.7)	19.1 (9.1)	7.4/ (7.1)
Completeness, %	96.6 (96.2)	99.9 (98.5)	98.4 (75.2)	100 (100)	100 (100)	100 (100)	98.7 (83.2)	99.6 (94.0)	99.7 (100)
Mean I/sigma	23.1 (2.4)	26.3 (1.1)	33.4 (1.6)	44.7 (4.0)	41.0 (4.4)	40.9 (6.3)	31.0 (0.67)	31.3 (1.5)	11.9 (2.4)
Wilson B‐factor, Å^2^	26.7	19.8	13.7	18.5	29.1	23.9	35.5	19.3	35.7
R‐merge	0.122 (0.843)	0.104 (0.928)	0.095 (0.980)	0.083 (0.943)	0.110 (0.974)	0.109 (0.692)	0.119 (0.898)	0.138 (0.901)	0.181/0.667
cc1/2 (highest resolution shell)	0.735	0.525	0.631	0.894	0.929	0.966	0.552	0.593	0.837
*R*_work_/*R*_free_	0.167/0.198	0.145/0.167	0.128/0.161	0.159/0.182	0.172/0.202	0.171/0.208	0.206/0.234	0.159/0.177	0.192/0.235
Protein residue ranges	A: 23–287, B: 23–288, C: 24–287, D: 23–288	A: 22–290	A: 23–288	A: 23–288	A: 23–288	A: 23–288	A: 23–288	A: 23–288	A: 23–288
Other atoms (water/the rest)	660/44	291/60	291/44	222/77	148/43	222/23		252/36	61/35
RMS bonds/angle	0.007/0.851	0.005/0.820	0.005/0.808	0.005/1.20	0.006/0.804	0.006/0.780	0.006/0.849	0.006/0.845	0.002/0.581
Ramachandran favored/outlier, %	97.1/0.0	96.3/0.0	96.6/0.0	95.8/0.0	95.8/0.0	97.0/0.0	96.2/0.0	97.7/0.0	96.2/0.0
Mean B‐factor, Å^2^ protein/ligand/water	34.9 34.5/44.2/38.4	24.1 22.4/36.5/33.9	18.1 16.1/20.8/32.0	23.5 21.6/51.4/32.6	33.8 33.5/40.2/37.1	26.8 26.0/39.0/32.6	42.3	23.2 23.4/31.2/33.1	36.5 36.2/53.7/37.2
http://firstglance.jmol.org/fg.htm?mol=PDBID	6U0Y	6U10	6U13	6U0Z	6UAH	6UAF	6UA1	6UAC	6U2Z

Statistics for the highest resolution shell are shown in parentheses.

**Table 4:** Melting temperature shift by metal ions

		Cd	Zn	Ni	Co	Cu	Mg	Mn
	Tm (°C)	dT (°C)						
NDM‐1	41	10	15	5	7	−0.5	0	0
L1	37	16.5/11.5	16/10	11	17.5	0	0	1.5

Tm, melting temperature of protein without metal ions; dT, temperature shift after addition of metal ions.

### 4.5 Structure determination

The structures were determined by MR using molrep implemented in the HKL3000 software package.^55^ At first, the L1‐Native structure was solved by MR using the structure of L1 (PDB ID http://firstglance.jmol.org/fg.htm?mol=5DPX) as the search model, and the rest of the structures were determined by using the L1‐Native structure as the search model. The initial models were manually adjusted using COOT^56^ and were then iteratively refined using COOT, PHENIX,^57^ and REFMAC.^58^ Initially REFMAC refinement worked better for the structure, which was then moved to PHENIX to finalize the refinement. Throughout the refinement for the structures, the same 5% of reflections were kept out throughout from the refinement (in both REFMAC and PHENIX refinement).

The final structures converged to reasonable *R*_work_ and *R*_free_ with regards to each resolution limit of all corresponding data. The stereochemistry of the structures was checked with PROCHECK^59^ and the Ramachandran plot and validated with the PDB validation server. Except for L1‐Native, which has four copies of the protein (residues 17–290) in the asymmetric unit, all have one protein chain and the ligand (Table 3). The protein chain contains an N‐terminal three residue cloning artifact (residues 17–19; Ser‐Asn‐Ala), which is produced after purification and cutting by the TEV protease, but some are apparently disordered. The final L1‐Native structure with *R*_work_/*R*_free_ of 17.6/19.8% includes four protein chains, each containing two zinc ions, and a total of nine ethylene glycol and 660 water molecules in the asymmetric unit. Similarly, L1‐Capt structure has one protein chain, two zinc ions, two captopril molecules, three formate molecules, one ethylene glycol and 291 water molecules; L1‐Mox has one protein chain, two zinc ions, a hydrolyzed moxalactam, three ethylene glycol molecules, and 290 water molecules; L1‐PenG has one protein chain, two zinc ions, a hydrolyzed penicillin G, a succinate, a bis‐tris, a sulfate, polyethylene glycol (PEG), one additional zinc ion and 226 water molecules; L1‐Imip has one protein chain, two zinc ions, a hydrolyzed imipenem, and 222 water molecules; L1‐Mero has one protein chain, two zinc ions, a hydrolyzed meropenem, a PEG, an ethylene glycol, and 149 water molecules. Ethylene glycol, PEG, sulfate, succinate, and bis‐tris molecules are originated from crystallization and/or cryo‐solution.

The structures of the metal‐free form and the complex with cadmium or copper ions were determined and refined by the same procedure. The final L1‐E structure (1.80 Å) includes the protein chain, one ethylene glycol molecule, and 107 water molecules. The L1‐Cd‐Mox‐E (1.60 Å) structure contains the protein chain, hydrolyzed moxalactam, two cadmium ions, and 273 water molecules; and L1‐Cu‐Mox‐E (2.38 Å) contains the protein chain, hydrolyzed moxalactam, two copper ions, one ethylene glycol molecule, and 52 water molecules. Coordinates of the structures have been deposited in the Protein Data Bank and the PDB IDs are shown in Table 3. Crystallographic data and refined model statistics are presented in Table 3.

### 4.6 Protein thermal shift assay

Samples of NDM‐1 and L1 were diluted to 250 μM in a buffer containing 20 mM Tris pH 7.5, 100 mM NaCl, 1 mM TCEP. Zinc ions were removed from the protein samples by incubating with 5 mM EDTA for 30 min and then dialyzing three times over 16 hr at 4°C using the buffer solution of 20 mM Tris pH 7.5, 100 mM NaCl, 1 mM TCEP. For the experiment, to 10 μM each of the metal‐depleted NDM‐1 or L1, 100 or 500 μM selected metals (ZnCl_2_, CdCl_2_, MgCl_2_, NiCl_2_, CoCl_2_, CuCl_2_, and MnCl_2_) and SYPRO Orange dye (Invitrogen) were added in a buffer 20 mM Tris pH 7.5, 100 mM NaCl, 1 mM TCEP. DSF of 50 μl samples on a 96‐well PCR plate (BioRad) was done on CFX Connect Real‐Time System (BioRad). Software parameters were lid temperature 105°C, sample equilibration 20°C for 30 min, and melting curve fluorescence signal acquisition from 25 to 90°C with ramp rate 1°C per 60 s. The experiment was done in triplicate and the resulting numbers were averaged.

### 4.7 Isothermal titration calorimetry

For the ITC assay, 15 μM each of NDM‐1 and L1 in 20 mM Tris pH 7.5, 100 mM NaCl, 1 mM TCEP buffer were prepared with 2 mM ZnCl_2_ or 1 mM EDTA. The protein samples, L1 with Zn or EDTA and NDM‐1 with Zn or EDTA, were titrated with 1 mM moxalactam in the same buffer solution using VP‐ITC calorimeter (MicroCal) with Origin7 and VPViewer 2000 software at 25°C with the reference power of 15 μcal/s. For the control, the buffer solution without antibiotics was used. The ITC experiment was carried out in single injection mode with injection parameters of volume 42.5 μl, duration 300 s, and spacing 1800 s. After injection, the molar ratio between protein and moxalactam was 1:2. The experiment was done in triplicate and the resulting numbers were averaged.

### 4.8 Enzymatic activity assay

To study the activity of purified MBLs in the presence of different metals ions, 400 μM protein samples of NDM‐1 and L1 were treated with 5 mM EDTA for 2 hr. The EDTA‐treated protein samples were diluted to 2 μM in a buffer containing 20 mM Tris pH 7.5, 100 mM NaCl, 1 mM TCEP, then incubated for 20 min with 250 μM of each selected metal (ZnCl_2_, CdCl_2_, MgCl_2_, NiCl_2_, CoCl_2_, CuCl_2_, and MnCl_2_). Each enzyme + metal sample was 10‐fold diluted into 0.3 mM meropenem (PHR1772, Sigma Aldrich) in the buffer containing 20 mM Tris pH 7.5, 100 mM NaCl, 1 mM TCEP, and 10 μM EDTA. After 1 min of incubation, absorbance was measured using NanoDrop 1000 (Thermo Scientific) spectrophotometer. The experiment was done in triplicate and the resulting numbers were averaged.
